# Clinical Characteristics and Low Vision Rehabilitation Methods for Partially Sighted School-Age Children

**DOI:** 10.4274/tjo.82653

**Published:** 2015-04-05

**Authors:** Zuhal Özen Tunay, Deniz Çalışkan, Aysun İdil, Derya Öztuna

**Affiliations:** 1 Zekai Tahir Burak Women’s Health Training and Research Hospital, Ophthalmology Clinic, Ankara, Turkey; 2 Ankara University Faculty of Medicine, Department of Public Health, Ankara, Turkey; 3 Ankara University Faculty of Medicine, Low Vision Rehabilitation and Research Center, Department of Ophthalmology, Ankara, Turkey; 4 Ankara University Faculty of Medicine, Department of Biostatistics, Ankara, Turkey

**Keywords:** low vision, low vision rehabilitation, school-age children

## Abstract

**Objectives:**

To determine the clinical features and the distribution of diagnosis in partially sighted school-age children, to report the chosen low vision rehabilitation methods and to emphasize the importance of low vision rehabilitation.

**Materials and Methods:**

The study included 150 partially sighted children between the ages of 6 and 18 years. The distribution of diagnosis, accompanying ocular findings, visual acuity of the children both for near and distance with and without low vision devices, and the methods of low vision rehabilitation (for distance and for near) were determined. The demographic characteristics of the children and the parental consanguinity were recorded.

**Results:**

The mean age of children was 10.6 years and the median age was 10 years; 88 (58.7%) of them were male and 62 (41.3%) of them were female. According to distribution of diagnoses among the children, the most frequent diagnosis was hereditary fundus dystrophies (36%) followed by cortical visual impairment (18%). The most frequently used rehabilitation methods were: telescopic lenses (91.3%) for distance vision; magnifiers (38.7%) and telemicroscopic systems (26.0%) for near vision. A significant improvement in visual acuity both for distance and near vision were determined with low vision aids.

**Conclusion:**

A significant improvement in visual acuity can be achieved both for distance and near vision with low vision rehabilitation in partially sighted school-age children. It is important for ophthalmologists and pediatricians to guide parents and children to low vision rehabilitation.

## INTRODUCTION

The main goals of visual habilitation and rehabilitation for children with low vision are developing visual perception, increasing quality of life by maximizing their existing sight using appropriate methods and helping them use this level of vision optimally, and providing these children equal opportunities in both education and social contexts.^1,2^

In a 2002 congress held in Australia by the International Council of Ophthalmology, use of the following terminology regarding low vision was recommended:^3,4^

Blindness: Conditions involving complete loss of visual function, where the individual can only be rehabilitated with vision substitution methods.

Low Vision: Conditions with lesser degrees of vision loss, where the individual benefits from vision enhancement devices.

The World Health Organization (WHO) bases its legal definitions of low vision and blindness on visual acuity and visual field. Low vision is thus defined as visual acuity in the better eye after refractive correction between 20/70 (0.3) and 20/400 (0.05, 3 mps) or a visual field less than 20 degrees. Blindness is defined as visual acuity less than 20/400 (0.05, 3 mps) in the better eye after refractive correction or a visual field less than 10 degrees.^3,4^

These boundaries are especially important in terms of determining the legal rights given to individuals with visual impairment. However, these arbitrary limits are not as important as an individual’s life goals when determining the need for visual rehabilitation. The decision to pursue low vision rehabilitation is not made according to legal limits, but is made individually based on that individual’s vision requirements and life goals.^5,6^

Based on WHO data from 2010, there are an estimated 1.5 million blind children and 5 million children with low vision worldwide. Visual impairment significantly affects the development and education of an estimated 1.5 to 2 million children. In Turkey, the disability rate is 12.58%, with the visually impaired accounting for 8.4% of the total disabled population. Approximately 32% of the Turkish population is under the age of 18, and 44% is under the age of 25. Using these data, the estimated number of visually impaired individuals in the child and adolescent age group in Turkey is about 350,000.^7^

The purpose of this study was to determine the diagnosis distribution and clinical characteristics of school-age children presenting for low vision rehabilitation services, to share methods chosen for low vision rehabilitation, and to emphasize the importance of rehabilitation in children with low vision.

## MATERIALS AND METHODS

The study included a total of 150 children with low vision attending the Ankara University Department of Public Health, Vision Rehabilitation and Research Center between 1 April 2012 and 28 February 2013.

The study was conducted in accordance with the Declaration of Helsinki. Approval to conduct the study was granted by the Ankara University Ethics Committee. Written informed consent was obtained from all children included in the study and their legal guardians.

Each child’s diagnosis, accompanying ocular findings, distance and near visual acuity with and without a low vision aid (LVA), and the type(s) of LVA used for distance and near vision were determined. Demographic characteristics of the children in the study and the consanguinity of their parents were recorded.

Refractive error was assessed with autorefractometer and retinoscope prior to visual acuity examination. Values for corrective lenses for optimal distance vision were determined taking into account the smallest increase in visual acuity value noticeable by each child. First, distance vision was determined using LogMAR scales in normal lighting conditions from a distance of 4 meters. For children with low visual acuity, the examination was repeated from 2 meters and 1 meter. Children’s near vision was then assessed using MNREAD cards at a distance of 25 cm.

Each child’s required magnification power was calculated based on Kestenbaum’s formula (1/visual acuity=dioptric power [1/VA=D]), then adjusted according to the individual and his/her desired visual function to obtain the final magnification power. Near and distance visual acuity with the resulting LVA was recorded. For low vision children with photophobia, lenses filtering the appropriate wavelengths based on the diagnosis were used.

The presence of strabismus was evaluated using a cover test, cover-uncover test and alternating cover test. In patients with deviations, an alternate prism cover test was used to measure the degree of deviation. Binocular vision was evaluated using the Worth 4 dot test. Anterior and posterior segment examinations were performed using a biomicroscope and binocular indirect ophthalmoscope.

The data were analyzed using Statistical Package for the Social Sciences version 11.0 for Windows (SPSS Inc., Chicago, USA) statistical software. Data are expressed as minimum (min), maximum (max), mean, standard deviation (SD), number or percent (%). Visual acuity with and without the use of an LVA was compared using a paired samples t test. P values less than 0.05 were accepted as statistically significant.

## RESULTS

The mean age of the children included in the study was 10.6±3.0 years and the median age was 10 years (range, 6-18 years). There were 88 (58.7%) males and 62 (41.3%) females; 45 (30.0%) of the children had been born in Ankara and 52 (34.7%) were living in Ankara at the time. The families of 7 of the children who had previously lived elsewhere stated that they had moved to Ankara to accommodate their child’s need for special education and rehabilitation.

The children living outside of Ankara came primarily from the Central Anatolia (14.6%) and Black Sea (14%) regions, while the fewest came from the Aegean (4.7%) and Eastern Anatolia (5.3%) regions (Table 1).

Analysis of the distribution of the children’s diagnoses revealed that the most common were hereditary vision impairment with 36% and cortical vision impairment with 18%. The distribution of the subjects’ diagnoses is shown in Table 2. The most frequent accompanying ocular findings were nystagmus (35.3%) and strabismus (30.0%).

Investigation of parental consanguinity revealed a 66% rate of consanguineous marriage, with 29.3% of the parents being first degree relatives, 24.0% second degree relatives, and 12.7% more distant relatives.

The most commonly employed vision enhancement aids were telescopic lenses (91.3%) for distance and magnifiers (38.7%) for near vision. The second most common near vision rehabilitation method was telemicroscopic systems, with 26.0% (39 subjects). Electro-optical devices were used by 5 (3.3%) children for distance and 6 (4.0%) for near vision. Filters were used by 14% of the children, 66.7% of whom were being followed with a diagnosis of albinism (Table 3).

The subjects’ near and distance visual acuities with and without vision enhancement aids and devices are shown in Table 4. LVA usage increased the mean distance visual acuity from 1.02 logMAR to 0.26 logMAR and provided an improvement in mean near visual acuity from 4.2 M to 1.38 M. The differences were statistically significant for both near and distance vision (paired samples t test, p=0.001).

## DISCUSSION

This study investigated clinical characteristics and low vision rehabilitation methods in school-age children with low vision. This type of study conducted with individuals presenting to clinics is advantageous over studies that are population-based or conducted in schools for the blind because they include more detailed ophthalmologic data.^8^ However, a major disadvantage is that the data cannot be generalized to the entire population. The Ankara University Low Vision Rehabilitation and Research Center is a university-based center that serves patients from every region of Turkey. Therefore, although data in this context may not reflect the general population, we believe they will contribute both in terms of referring children with low vision to rehabilitation services and to the planning and implementation of low vision rehabilitation services.

It has been reported in the literature that male patients in both the pediatric and adult age groups present more frequently for low vision rehabilitation.^3,9,10,11^ Consistent with the literature, the gender distribution in our study group was 58.7% male and 41.3% female. In a study conducted by Cardiff University in the United Kingdom, 67% of the children were male.^12^ In another study by Gothwall and Herse10 including children between the ages of 8 and 18 years with low vision living in India, 55% of the patients presenting for rehabilitation were male.

Forty-five (30%) of the children in the current study were born in Ankara, and 52 (34.7%) were living in Ankara at the time of the study, indicating that approximately 2 out of 3 children in our study were coming from outside Ankara. There were patients from each of the seven geographical regions of Turkey, with the highest proportion coming from the Central Anatolia and Black Sea regions, and the lowest proportion from the Aegean region. According to data from the Turkish Statistical Institute (TSI), the Aegean and Marmara regions have the lowest rates of consanguineous marriage.^7^ The smaller number of low-vision children from the Aegean region compared to the other regions may be attributable to this. However, the Southeastern and Eastern Anatolia regions of Turkey, which have the highest rates of consanguineous marriage (43%), were not most represented in our study group. This demonstrates the need to consider various other factors including differences in economic development, transportation difficulties and distance from Ankara. Furthermore, individuals with low vision living in the Aegean and Marmara regions have access to local low vision rehabilitation services, which we believe may also contribute to the lower number of patients presenting from these regions.

The families of seven children stated that they had relocated to Ankara in order to accommodate their child’s special educational and rehabilitation needs. This indicates a need for low vision rehabilitation centers in the other regions and provinces of Turkey. According to the WHO, a low vision rehabilitation center is required for every 10 million of population,^1,3^ meaning there is a need for 6 additional centers in Turkey.

Analysis of the distribution of the diagnoses of the visually impaired children in our study revealed that the most common diagnosis was hereditary macular dystrophy (36%), followed by cortical visual impairment (18%). Other common diagnoses, in order of frequency, were albinism, optic atrophy, structural anomalies, retinitis pigmentosa and retinopathy of prematurity. Olusanya et al.^8^ published a study last year evaluating the patient profile in Nigeria’s only low vision rehabilitation clinic over a period of 3 years and reported that 45 children between the ages of 0 and 15 years presented to the clinic, with the most common causes being optic atrophy (24.4%) and albinism (24.4%). Indian researchers Gothwall and Herse10 reported that the most common diagnoses they found were retinal conditions, primarily heredomacular degeneration (21.5%) and retinitis pigmentosa (19.6%), followed by structural causes (12.0%) and albinism (5.0%). In a Turkish study by İdil1,^11^ evaluating visually impaired children between 2004 and 2009, the leading diagnoses among those aged 7-18 were heredomacular degeneration (42%), albinism (21%) and optic atrophy (13%). In European, American and Australian studies, the most common diagnoses are retinopathy of prematurity (12-28%) and cortical causes of blindness (25-30%), while visual impairment due to hereditary causes is rarely encountered.^3,13,14,15^

Parental consanguinity is believed to be the leading cause of the prominence of hereditary conditions in children in Turkey. According to 2010 TSI data, the rate of consanguineous marriage in Turkey is 21%. This rate increases to 35% in Southeastern Anatolian provinces, and is lowest in Western Anatolia at 12-14%. Compared to the rate of 0.2-2% found in European countries, the proportion of consanguineous marriages in Turkey is extremely high.^16,17^ Parental consanguinity increases the incidence of some hereditary diseases such as congenital cataract, retinitis pigmentosa, and congenital glaucoma up to 50 fold.^11,18^

Following hereditary causes, the most common diagnoses in this study were cortical visual impairment (18%), optic atrophy (10%) and retinopathy of prematurity (4.7%). A comparison with previous studies conducted by Turan et al.^19^ and İdil^11^ in 2002 and 2011, respectively, indicates that the frequency of vision loss due to these diagnoses has increased. This may be attributable to the development and wider availability of neonatal intensive care, which has allowed the survival of more premature newborns overall and of those with lower birth weight. Morbidities associated with prematurity include hydrocephaly and periventricular leukomalacia, as well as conditions resulting from these pathologies such as optic atrophy, cortical atrophy and retinopathy of prematurity due to incomplete retinal development.^20^

The most common vision enhancement aids utilized by the visually impaired children included in this study were telescopic glasses (91.3%) for distance and magnifiers (38.7%) for near vision. The second most common method for near vision rehabilitation was telemicroscopic systems (21.6%). Electro-optical devices were utilized by 5 children (3.3%) for distance and 6 children (4.0%) for near vision. Telescopic systems are chosen more often because they are more economical and portable than electro-optical systems. Similarly, magnifiers are most popular for near vision because they are economical and effective systems to which low vision patients, especially those newly starting rehabilitation, can adapt easily. However, for individuals with very low visual acuity (severe low vision), better results in both distance and near vision can be achieved with electro-optical systems. Mosuro et al.^21^ screened low vision children attending schools for the blind in Nigeria and reported that telescopic systems were most commonly utilized for distance (94%), while magnifiers were used most often for near vision (69%). Similar results were reported in studies by Margrain^22^ in the United Kingdom and DeCarlo et al.^23^ in the United State of America in 2000 and 2012, respectively.

Filters were used by 14% of the low vision children included in this study, 66.7% of whom were being followed for albinism. In a 2013 study, Palomo-Álvarez and Puell^24^ reported that special filters were effective in alleviating photophobia in hereditary fundus dystrophy and albinism but did not significantly improve reading performance. For the children in their study, filters were utilized to lessen photophobia and improve distance vision.

Comparing the subjects’ near and distance vision levels to those achieved with vision enhancement aids, mean distance vision level increased from 1.02 logMAR to 0.26 logMAR and near vision improved from 4.20 M to 1.38 M with LVA use. The differences were significant for both near and distance (p=0.001). Low vision rehabilitation resulted in marked improvements in the vision levels of the visually impaired children included in this study.

## CONCLUSION

In conclusion, low vision rehabilitation can facilitate significant improvement in both near and distance vision in visually impaired school-age children. Therefore, it is crucial that both pediatricians and ophthalmologists refer children with visual impairment to vision rehabilitation.

## Ethics

Ethics Committee Approval: Ankara University Ethics Committee, Informed Consent: Patient informed consent present.

Peer-review: Externally peer-reviewed.

## Figures and Tables

**Table 1:**
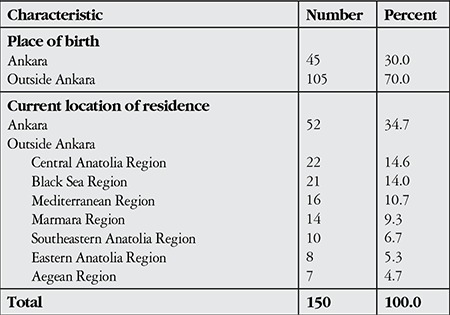
Distribution of the children with low vision included in the study based on the locations of their birth and residence

**Table 2:**
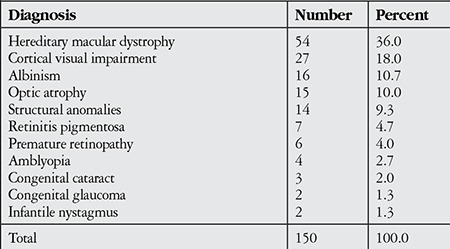
Distribution of the diagnoses of the children with low vision included in the study

**Table 3:**
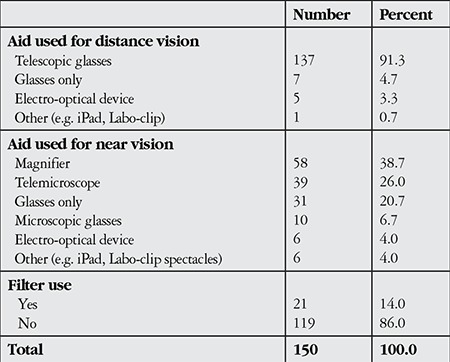
Distribution of low vision children included in the study by low vision rehabilitation device usage

**Table 4:**
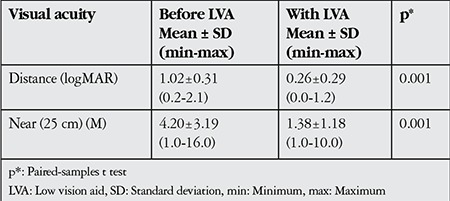
Comparison of visual acuity levels of the study subjects before and while using low vision aids
